# Three-dimensional continuous picking path planning based on ant colony optimization algorithm

**DOI:** 10.1371/journal.pone.0282334

**Published:** 2023-02-27

**Authors:** Chuang Zhang, He Wang, Li-Hua Fu, Yue-Han Pei, Chun-Yang Lan, Hong-Yu Hou, Hua Song

**Affiliations:** School of Mechanical Engineering and Automation, University of Science and Technology Liaoning, Anshan, China; Dai Hoc Duy Tan, VIET NAM

## Abstract

Fruit-picking robots are one of the important means to promote agricultural modernization and improve agricultural efficiency. With the development of artificial intelligence technology, people are demanding higher picking efficiency from fruit-picking robots. And a good fruit-picking path determines the efficiency of fruit-picking. Currently, most picking path planning is a point-to-point approach, which means that the path needs to be re-planned after each completed path planning. If the picking path planning method of the fruit-picking robot is changed from a point-to-point approach to a continuous picking method, it will significantly improve its picking efficiency. The optimal sequential ant colony optimization algorithm(OSACO) is proposed for the path planning problem of continuous fruit-picking. The algorithm adopts a new pheromone update method. It introduces a reward and punishment mechanism and a pheromone volatility factor adaptive adjustment mechanism to ensure the global search capability of the algorithm, while solving the premature and local convergence problems in the solution process. And the multi-variable bit adaptive genetic algorithm is used to optimize its initial parameters so that the parameter selection does not depend on empirical and the combination of parameters can be intelligently adjusted according to different scales, thus bringing out the best performance of the ant colony algorithm. The results show that OSACO algorithms have better global search capability, higher quality of convergence to the optimal solution, shorter generated path lengths, and greater robustness than other variants of the ant colony algorithm.

## Introduction

The fruit-picking process uses the robot’s multi-sensor fusion technology to fuse the information obtained from multiple sensors to find the path from the robot’s initial position to the known target fruit point location. Enables the picking robot to move safely from the initial position to the target fruit point position [1]. Since the planning of the fruit picking path plays a decisive role in fruit picking efficiency, this paper focuses on the planning of the continuous picking path of the picking robot to improve the picking efficiency of the picking robot.

To solve this problem, researchers have proposed three types of planning algorithms: traditional algorithms, meta-heuristic algorithms, and deep learning. Among the traditional algorithms are the artificial potential field method(APF) [2], rapidly exploring random tree (RRT) [3], etc. Meta-heuristic algorithms include the ant colony algorithm [4], particle swarm algorithm [5], and gray wolf algorithm [6]. From the aspect of traditional algorithms, Liu et al. [7] proposed a time-optimal rapidly-exploring random tree (TO-RRT) algorithm for the path planning of citrus picking robots. The method controls the target offset probability of the random tree through the potential field. It introduces a node first search strategy and a regression superposition algorithm to enable the random tree to quickly escape from the repulsive potential field and enhance the ability of the random tree to explore the unknown space in the repulsive potential field. The method improves the robotic path planning speed by 99.73%, reduces the path length by 17.88%, and reduces the number of collision detections by 99.08%. Cao et al. [8] proposed an improved fast exploratory random tree (RRT) algorithm to solve the path planning problem for litchi-picking robots. The method adopts the idea of target gravity to speed up the path search and uses genetic algorithm and smoothing to optimize the path generated by RRT. The experimental results show that the collision-free path planned by the algorithm can successfully drive the manipulator from the initial position to the target position without collision. Xie et al. [9] used the artificial potential field method to plan and simulate the apple picking path, transferred the principle of applying two-dimensional potential in artificial potential field to three-dimensional space, obtained a smoother obstacle avoidance motion curve, and realized the picking path design according to the obstacle avoidance case and non-obstacle avoidance case respectively. The results show that picking path planning based on artificial potential fields can effectively avoid obstacles and achieve timely and reliable picking. Tang et al. [10] proposed a new method to improve the APF mathematical model and calculate the attractive moments directly in the joint space for path planning of the picking manipulator. Compared with the active filter, the computation time and the total joint error are reduced by 54.89% and 45.41%, respectively, using the latter method. Taking a citrus picking robot as an example, the new algorithm was verified in the offline design phase and the online actual picking test phase, respectively. Huang et al. [11] proposed a headland turning method for an autonomous picking robot. Three steps were executed during headland turning. First, end-of-line detection is based on machine vision. Second, the designed fast attitude adjustment algorithm based on satellite information is utilized. Third, a curve trajectory tracking controller is designed for turn control. The results show that the designed steering method enables the robot to converge to the path faster and stay on the path with less radial error, thus reducing the time, space, and deviation of the ground steering and thus improving the efficiency of the fruit-picking robot. From the aspect of metaheuristic algorithm, Yan et al. [12] proposed an improved multi-objective particle swarm optimization algorithm (represented as GMOPSO) to solve the fruit picking problem. The algorithm combines variational operators, annealing factors, and feedback mechanisms to improve the diversity of populations, avoid local optimal solutions and speed up convergence while satisfying stable motion. The experimental results show that the robotic trajectory obtained by the GMOPSO algorithm can effectively complete fruit picking with an average picking time of 25.5 s and a success rate of 96.67%. Yuan et al. [13] proposed an improved picking path optimization method. In this paper, the coordinates of ripe apples were obtained by binocular cameras, the picking path planning problem was viewed as a three-dimensional TSP problem, and the best picking path was obtained using the improved ant colony algorithm. Kiani et al. [14] finds the collision-free optimal path between two points for the robot by extending the gray wolf optimization (ex-gwo). The optimal path cost of 55.56% based on the ex-gwo algorithm has a good success rate. From the aspect of deep learning, Lin et al. [15] used a fast collision-free path planning method based on deep reinforcement learning to solve the pomegranate picking problem. A recurrent neural network is first used to memorize and utilize the past states observed by the robot. Then a deep deterministic policy gradient algorithm (DDPG) is used to predict the collision-free path of the states. The results show that the method can plan a collision-free path with a high success rate in a shorter time. Liu et al. [16] proposed a reinforcement learning strategy incorporating expert experience guidance when applying deep reinforcement learning algorithms to the path planning of a multi-degree-of-freedom fruit-picking manipulator in an unstructured environment. The experimental results show that the method can effectively improve the model’s performance in an unstructured environment and increase the learning efficiency and path planning success rate at the early stage of training. Li et al. [17] focused on the problem of visual recognition and path planning for intelligent fruit-picking robots. They established a stereo vision-based identification and positioning system for picking robots, and the coordinate error of the target point of the intelligent fruit picking robot coordinate system is less than 10 mm, with high accuracy. Then, path planning is performed for the intelligent fruit-picking robot based on visual feedback algorithm and biostimulation neural network. The results show the effectiveness of the intelligent approach. Yang et al. [18] proposed a Residual-like Soft Actor Critic (R-SAC) algorithm for agricultural scenarios to realize safe obstacle avoidance and intelligent path planning of robots. In addition, they proposed an offline expert experience pre-training method and optimized the reward mechanism of the algorithm using multi-step TD error. The experimental results show that the method has stable performance in static and dynamic obstacle environments and outperforms other reinforcement learning algorithms. Wagner et al. [19] proposed a compact and efficient network architecture for estimating the orientation of soft fruits, such as the color and depth images of strawberries. The picking robot is made aware of the complete pose of the crop or fruit to be collected to perform proper path and collision planning. The system can be trained automatically in a realistic simulation environment. Depending on the amount of training data used, the coverage of the state space, and the availability of RGB-D or RGB data only, average errors as low as 11° could be achieved.

However, the existing research on picking robots is mainly in the direction of single path planning, i.e., the path planning for fruit picking must be re-programmed after each picking session. The selection problem for continuous picking needs to be better-studied. There is a problem of high workload and low efficiency when the number of fruits is high, which also reduces the overall picking efficiency. Therefore, it is essential to investigate the optimal path for continuous picking of the picking robot.

To this end, this paper abstracts the continuous picking path planning problem as a three-dimensional Traveling Salesman Problem (TSP) solved with the shortest path as the objective without considering obstacle avoidance conditions. Compared with other algorithms, the metaheuristic algorithm has the advantages of simplicity, efficiency, and adaptability, so it is widely used in path planning. Among these metaheuristics, the ant colony algorithm, as an intelligent search algorithm, was proposed by the Italian scholar DORIGO as an intelligent bionic algorithm by simulating the characteristics of ants’ foraging behavior and was later used to solve the travel quotient problem. Compared with other metaheuristic algorithms, ACO has the advantages of good self-organization, robustness, positive feedback, parallelism, and easy integration with different algorithms. Due to these advantages, the ant colony algorithm has been widely used in path planning.However, it still has some disadvantages, such as being easy to fall into local optimum, time-consuming, and challenging to get rid of deadlock. Therefore, the contributions of this paper are as follows:

1. The optimal sequential ant colony optimization algorithm is proposed to overcome the problems of premature ant colony algorithm, too fast convergence, and strong influence by parameters.
2. In this paper, the optimal sequential ant colony optimization algorithm is used to solve the picking manipulator’s three-dimensional continuous picking path planning problem. The effectiveness of the optimal sequential ant colony optimization algorithm was verified by comparing the effect of continuous picking path planning with the ant colony algorithm and two ant colony improvement algorithms.

The purpose of this paper is to propose an efficient solution to the little-studied path-planning problem of continuous fruit picking. The OSACO algorithm proposed in this paper solves the problems of early maturity, too fast convergence, and poorly planned paths due to the influence of parameters when the ant colony algorithm is used for path planning of continuous fruit picking. Through simulation, the OSACO algorithm can efficiently solve the path-planning problem of continuous fruit picking. The rest of this paper is organized as follows. In Section 2, the basic ant colony algorithm and the specific implementation of the optimal sequential ant colony optimization algorithm are introduced, respectively. Experimental simulations are performed in Section 3 to verify the superiority of the OSACO algorithm by comparing it with the ant colony algorithm and two ant colony improvement algorithms. The conclusions of this paper are presented in Section 4. The limitations and future works of this paper in Section 5.

## Ant colony algorithm

### Development of ant colony algorithm

Ant Colony Optimization (ACO) is a swarm intelligence algorithm proposed by Italian scholars DORIGO et al. [4] in 1996, which was first applied to the TSP problem. It was further optimized in 1997 by DORIGO et al., who proposed an ant colony system [20]. The algorithm introduces the concept of hitherto optimal in the pheromone update problem and incorporates local pheromone update rules, further improving the algorithm’s performance. Bullnheimer et al. [21] proposed a ranking-based ant colony algorithm, which allows only the top-ranked ants to secrete pheromones and gives the most robust feedback to the known optimal paths, allowing the performance of the algorithm to be further improved. Stützle et al. [22] proposed the maximal, minimal ant system algorithm, which improves the pheromone update method and the bounds of the traditional ant colony algorithm. Yang et al. [23] proposed an entropy learning strategy to optimize the accuracy of understanding by adaptively improving diversity and convergence and jumping out of local optimum by setting up a game strategy and introducing mean filtering. Li et al. [24] proposed a slime bacteria-ant colony fusion algorithm (SMACFA), which first obtains the initial planning path by SMA. Then, a high-quality pipe is selected from the paths obtained by SMA, and the two ends of the pipe are used as fixed-point pairs; Finally, the fixed-point pair is directly applied to the ACO by fixing the selection principle. The method fully validates that SMACFA outperforms the performance of other algorithms in solving TSP. Shahadat et al. [25] proposed an improved ACO-based approach called ACO with Adaptive Visibility (ACOAV). This method uses a new distance metric that incorporates partial updates of individual solutions and 3-Opt local search operations. By comparison, ACOAV generates better solutions compared to ACO while spending less computational time; Han et al. [26] proposed a hybrid symbiotic biological search (SOS) and ACO algorithm (SOS-ACO). After assigning specific parameters to the ACO, the remaining parameters can be optimized adaptively by SOS. Using the optimized parameters, the ACO finds the optimal or near-optimal solution.

### Principle of basic ant colony algorithm

At the initial moment, M ants are randomly placed into different nodes, and the initial information of all nodes is set in the standard ant colony algorithm. The ants determine the next node to which the ants go according to the state probability rule in Eq (1).

$$P_{ij}^{k}(t)=\left\{\begin{array}{cc} \frac{{\left[\tau_{ij}(t)\right]}^{\alpha }{\left[\eta_{ij}(t)\right]}^{\beta }}{\sum_{s\in allowed_{k}}{\left[\tau_{is}(t)\right]}^{\alpha }{\left[\eta_{is}(t)\right]}^{\beta }} & ,j\in allowed_{k}\\ 0 & otherwise \end{array}\right.$$ (1)

Where *allowed*_*k*_ is the ensemble of nodes allowed to be selected by the ant in the next step. *τ*_*ij*_ is the pheromone content on edge *i*, *j*. *η*_*ij*_ is the degree of inspiration when transferring between *i*, *j*. $P_{ij}^{k}(t)$ is the probability of the kth ant moving from node *i* to node *j* at moment *t* (The roulette wheel method is used to select the next picking node, and it does not necessarily go to the node with the highest probability value). *s* is a node in the set of nodes not yet visited. *α* is the information heuristic factor. *β* is the desired heuristic factor.

The way to measure the distance between adjacent nodes is the Euclidean distance, as follows:

$$D_{ij}=\sqrt{{\left(x_{j}-x_{i}\right)}^{2}+{\left(y_{j}-y_{i}\right)}^{2}+{\left(z_{j}-z_{i}\right)}^{2}}$$ (2)

The specific formula for calculating *η*_*ij*_ is as follows.


$$\eta_{ij}=1/D_{ij}$$ (3)

The rules for the release of pheromones during crawling in ants are:

$$\tau_{ij}(t+1)=(1-\rho )\tau_{ij}+\Delta \tau_{ij}$$ (4)

$$\Delta \tau_{ij}(t)=\left\{\begin{array}{cc} \frac{Q}{L_{k}(t)} & \begin{array}{cccccc} ,Ant & k & passed & through & path & i,j \end{array}\\ 0 & ,otherwise \end{array}\right.$$ (5)

where *ρ* is the pheromone volatility factor. Δ*τ*_*ij*_(*t*) is the pheromone released by the ant to path *L*_*ij*_ at time *t* in the current loop. *Q* is the pheromone volatilization coefficient. *L*_*k*_(*t*) is the length of the ant’s crawl in this loop.

## The optimal sequential ant colony optimization algorithm

In response to the shortcomings of ant colony algorithms, such as easy prematureness, stagnation, falling into local optimum, and being influenced by parameters. In this paper, the optimal sequential ant colony optimization algorithm is proposed to improve on the appeal deficiency by introducing the following aspects.

1. a new way of updating pheromones locally and globally.
2. qualifying pheromones and introducing reward and punishment mechanisms.
3. adaptive adjustment mechanism of volatile factors.
4. an improved genetic algorithm is used to optimize the initial parameters of this algorithm.

### A new pheromone global and local update approach

#### Global pheromone update method

In the basic ant colony algorithm, each ant releases pheromones in each round of iteration to provide a reference for the next ant to choose a path. And in this paper, only ants with path length ranking in the top *x*% are allowed to release pheromones with additional reinforcement, and a new way of pheromone update is given. And the solution of how to select the specific number of ants in the top *x*% of the path length ranking is given in combination with the genetic algorithm, and other adjustment mechanisms are introduced to improve the path planning effect. The number of good ants is Calculated by Eq (6):

ω=mx (6)

Where, *ω* is the number of good ants, *m* is the number of ants, and *x* is a random number between 0 and 100.

The pheromone update process starts by allowing only the top x% of ants to perform pheromone release, after which a reward and punishment mechanism is introduced for enhanced global pheromone update. After all ants have gone through one round of iterations, find the optimal solution (shortest path length) and the worst solution (longest path length) in this round of iterations. Then the global pheromone on the total path where the ant finds the optimal and worst solution is updated according to Eq (4):

$$\tau_{ij}(t+n)=(1-\rho )\tau_{ij}(t)+\omega \Delta \tau_{fw}$$ (7)

$$\Delta \tau_{fw}=\left\{\begin{array}{cc} Q/L_{\text{finest}} & ,E(i,j)\in L_{\text{finest}}\\ -Q/L_{weakest} & ,E(i,j)\in L_{weakest}\\ 0 & ,others \end{array}\right.$$ (8)

Where *L*_*finest*_ is the current global optimal path and *L*_*weakest*_ is the current worst path. Here, by introducing a reward and punishment system, the global optimal solution found by the current iteration is reinforced while weakening the influence of the worst solution on the ant’s path selection.

#### Local pheromone update method

In this paper, a new pheromone update rule is used. To avoid ants converging to the same path, this paper specifies that the local pheromone is updated after each loop. After all ants have completed one iteration, the global pheromone is updated according to Eqs (7) and (8) above. This method makes the difference of pheromones between the optimal path and other paths increasingly larger, which searches quickly focus on the range of solution sets of optimal and sub-optimal paths and dramatically improves the search efficiency.

The local pheromone is updated according to Eqs (9) and (10):

$$\tau_{ij}(t)=(1-\rho )\tau (i,j)+\sum_{k=1}^{\omega -1}\left(\omega -k)\Delta \tau_{k}(i,j\right)$$ (9)

$$\Delta \tau_{k}(i,j)=\left\{\begin{array}{cc} Q/L_{k} & \begin{array}{cccccc} ,Ant & k & passed & through & path & i,j \end{array}\\ 0 & ,otherwise \end{array}\right.$$ (10)

### Restricted pheromones

In the unimproved ant colony algorithm, ants will prefer the path with more pheromones accumulated before when they choose a path. The remaining paths will cause the algorithm to stall when no ants pass by for a long time and the pheromones are played out. In this paper, we introduce the simplified max-min ant idea [22], which is to fix the domain of the pheromone.


$$\tau \in \left[\tau_{\text{min}},\tau_{\text{max}}\right]$$ (11)

The initial value of the pheromone is *τ*_0_ = *x* ⋅ *τ*_max_. The method of pheromone domain fixation makes the difference between the pheromone values on each path not very large, which gives the ants more path selection rights and effectively prevents the algorithm from converging stagnant prematurely while speeding up the convergence to the optimal solution. And the specific values of *τ*_min_, *τ*_max_ here do not affect the algorithm’s performance.

### Volatile factor adaptive adjustment mechanism

*ρ* is the pheromone volatility parameter and takes values in the range [0,1]. It reflects the evolutionary state of the whole ant colony system, and its size is directly related to the ant colony algorithm’s global search ability and convergence speed. Traditional algorithms have been setting *ρ* to a constant value, and most of people use adaptive factor volatility to adapt to more environments and save the solution time of optimal solution. The essence of adaptive pheromone factor volatility is that giving a larger value of *ρ* at the beginning of the algorithm increases the diversity of path selection, improves the global search capability, and avoids premature convergence or stagnation. This value is gradually reduced as the number of iterations increases, and a smaller value of *ρ* is assigned later in the algorithm to reduce the search diversity and accelerate the algorithm’s convergence to the optimal path. Qian et al. [27] divides the iterative process into two evenly spaced segments, which are given different values in the pre-and post-iteration, thus achieving an adaptive effect. Wang et al. [28] and Liu et al. [29] are both improved in a more traditional way, introducing random numbers between 0 and 1 in the first stage, gradually decreasing the value by continuous iteration, and manually setting the minimum value in the later stage to achieve the effect of adaptive volatilization. Shao et al. [30] optimized on the basis of the appeal improvement approach, where a random number between 0 and 1 is no longer introduced upfront, but the ratio of the current iteration number to the maximum iteration number, achieving the effect of dynamic adjustment.

All the above adaptive improvement methods revolve around their substance to continuously improve the function, i.e., to seek out the function with monotonically decreasing properties between [0,1]. In this paper, we compare these improvements and finally adopt the adaptive adjustment mechanism of the pheromone volatility factor divided according to the number of iterations according to the experimental effect. And debug the adaptive parameters of the volatility factor that are more suitable for the 3D TSP problem, as shown in Eq (7):

$$\rho =\left\{\begin{array}{cc} 0.8 & 0<g\leq G_{\text{max}}/4\\ 0.6 & G_{\text{max}}/4<g\leq G_{\text{max}}/2\\ 0.4 & G_{\text{max}}/2<g\leq 3G_{\text{max}}/4\\ 0.25 & 3G_{\text{max}}/4<g\leq G_{\text{max}} \end{array}\right.$$ (12)

Where *g* is the current number of iterations; *G*_max_ is the maximum number of iterations initially set.

### Optimization of ant colony algorithm parameters based on improved genetic algorithm

In this paper, the initial parameters *α*, *β*, *Q*, *x* of the ant colony algorithm are optimized using genetic algorithm to improve the performance of the ant colony algorithm. The feasibility of the idea has been validated by Marcin et al. [31]. Dorian et al. [32] investigated the interdependence between the parameters of ant colony algorithms and verified the importance of parameter settings for fast convergence to the best-known solution. Mostafa et al. [33] extended new ideas on the TSP problem using a new hybrid approach based on particle swarm optimization, ant colony optimization, and 3-Opt algorithm to solve the travel quotient problem. Riabko et al. [34] elaborated on the application of the 3D TSP problem and verified that the ant colony algorithm remains effective in solving the 3D TSP problem.

In this paper, an improved ant colony algorithm is used to make it more effective for the 3D TSP problem, taking into account the effect of parameters on the ant colony algorithm. Combining the idea of genetic algorithm with ant colony system, the best performance of the algorithm can be achieved without setting parameters manually.

### Solving process

1. using binary encoding.
  The parameters *α*, *β*, *x*, *Q* are represented by 4 code length binary code strings *x*_1_, *x*_2_, *x*_3_, *x*_4_ respectively. It is then combined into a binary bit string representing a combination of parameters. Considering the research results of Luo et al. [35], Wang et al. [36], and Liu et al. [37] on the parameter selection of ant colony algorithm. The overall performance of the algorithm is best when *α* ∈ [1,3]. The algorithm has better performance in all aspects when *β* ∈ [5,9]. *Q* ∈ [10,10000].
2. Adaptability function.
$$fitness=\frac{1}{L_{finest}}$$ (13)
  Where *L*_*finest*_ is the global optimal path length found by the ant colony algorithm, and the objective and fitness functions are reciprocals of each other.
3. Generate the initial population.
  A certain number of initial groups are randomly generated, coded and brought into the improved ant colony algorithm, and the size of the fitness is the criterion to measure the merit of ants.
4. Genetic operators.
  Like the ant colony algorithm, the selection operation still adopts the roulette wheel method; the crossover and variation operations adopt the adaptive approach of Eqs (14) and (15), where the variation operation takes the multivariate bit approach.
$$P_{c}=P_{\text{c1}}+\frac{p_{c1}\left(f_{\text{max}}-f\right)}{f_{\text{max}}-f_{avg}}$$ (14)
$$P_{m}=P_{m1}+\frac{P_{m1}\left(f_{\text{max}}-f\prime \right)}{f_{\text{max}}-f\prime }$$ (15)
  where *P*_*c*_, *P*_*m*_ is the crossover and variance probability at the time of operation; *P*_*c*1_, *P*_*m*1_ is the initial crossover and variance probability. *f*_max_ is the maximum fitness value in the population. *f*_agv_ is the population mean fitness value. *f* is the higher fitness value of the two individuals to be crossed. *f*′ is the fitness of the variant individual.
5. Algorithm parameter setting.
  Improved ant colony algorithm parameter settings: number of ants *m* = 50, maximum number of iterations *G* = 300. Genetic algorithm parameters are set: population size *P* = 40, number of iterations is 200, crossover probability *P*_*c*1_ = 0.7, and variation probability *P*_*m*1_ = 0.03.

The specific implementation steps in the optimal sequence algorithm are as follows:

1. Select the coding method of the genetic algorithm, initialization parameters such as population size, hybridization rate, variation rate, the maximum number of iterations and the initialized number of iteration cycles.
2. The value of the fitness function was calculated for each individual in the population according to Eq (13).
3. Perform genetic manipulations (selection, hybridization, and mutation) according to Eqs (14) and (15).
4. Determine whether the algorithm converges and whether the number of cycles exceeds the set maximum number of iterations. If either of these conditions is met, the algorithm converges. Otherwise, the genetic operation continues.
5. The part of the optimal solution generated by the genetic algorithm in step 4 with the highest fitness value is converted into part of the initialization parameters of the ant colony algorithm.
6. Initialize each parameter in the ant colony algorithm, such as the number of iterations *g* and the number of ants in colony *m*.
7. The initial position of ants is randomly placed, and the subsequent moving node of each ant is selected according to Eq (1).
8. Step 7 is executed for all ants, comparing the size between the current searched path length and the optimal path length. If the current path distance is small, then the optimal path is updated.
9. Update the pheromone values by Eqs (7) and (9). And save all path information.
10. When all ants have completed one round of iterations, reset the forbidden table and reinitialize the positions of only ants.
11. If the number of cycles reaches the set maximum, the algorithm ends; otherwise, skip to step 8.

**Fig 1:**
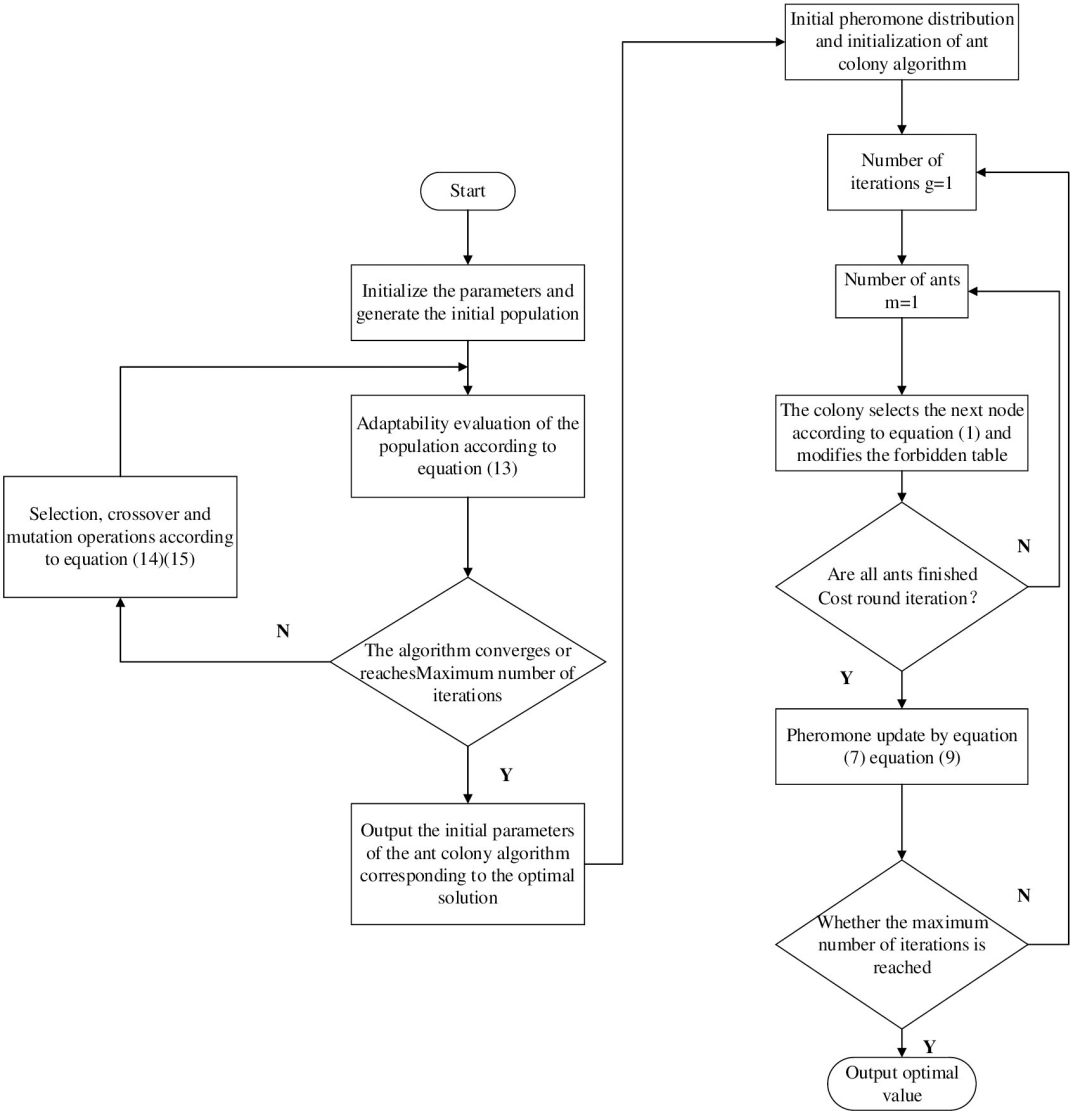
Optimal sequence ant colony algorithm implementation flow block diagram.

According to the implementation steps of the above algorithm, the implementation steps are presented in the form of a flow chart, as shown in Fig 1. The flow chart for the specific implementation of the optimal sequence algorithm is as follows:

## Simulation and results analysis

**Fig 2:**
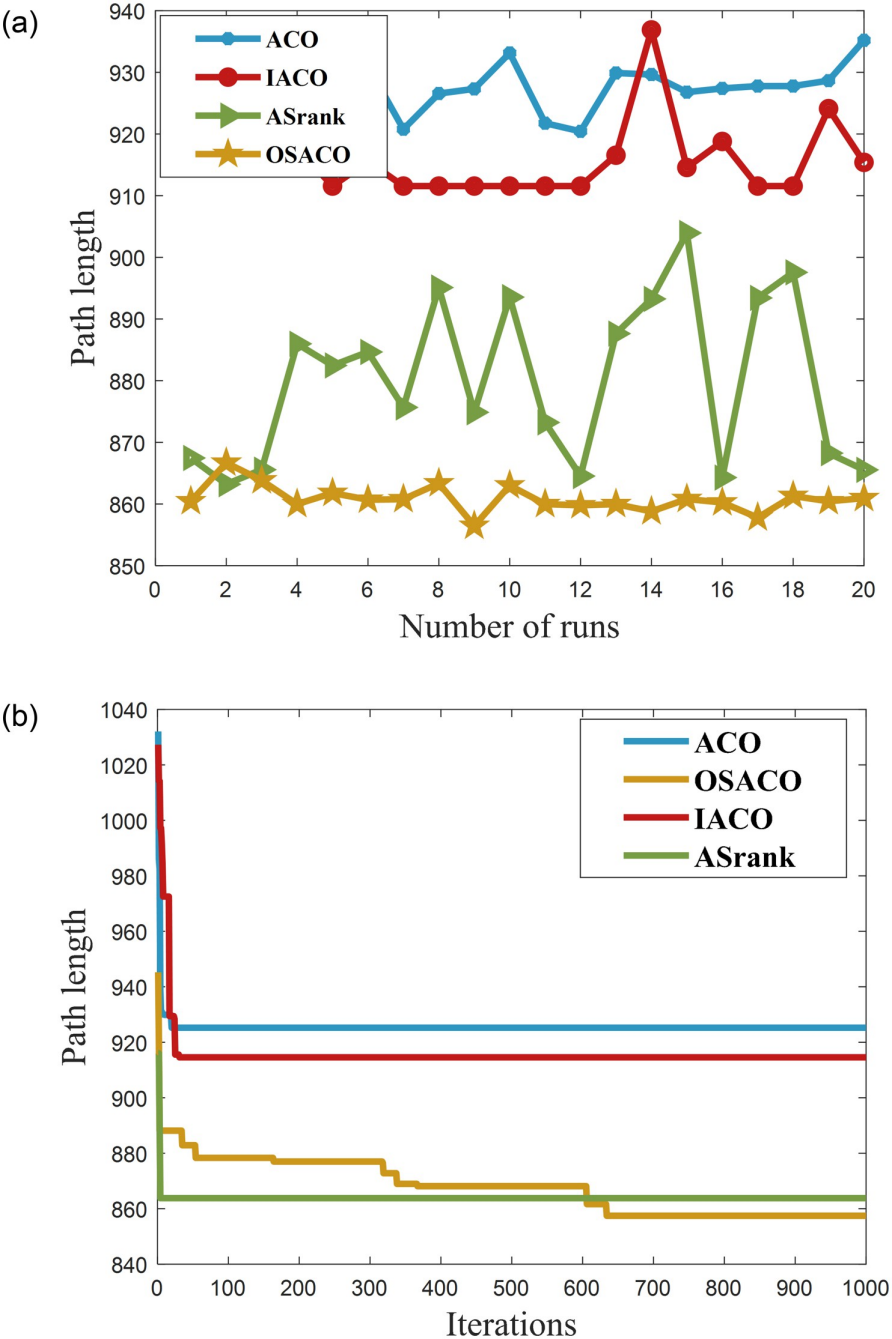
Number of independent runs and convergence curves of the four algorithms at 40 fruit numbers. (a) Four algorithms run independently for 20 times result graph. (b) Convergence curves under the optimal path conditions.

**Fig 3:**
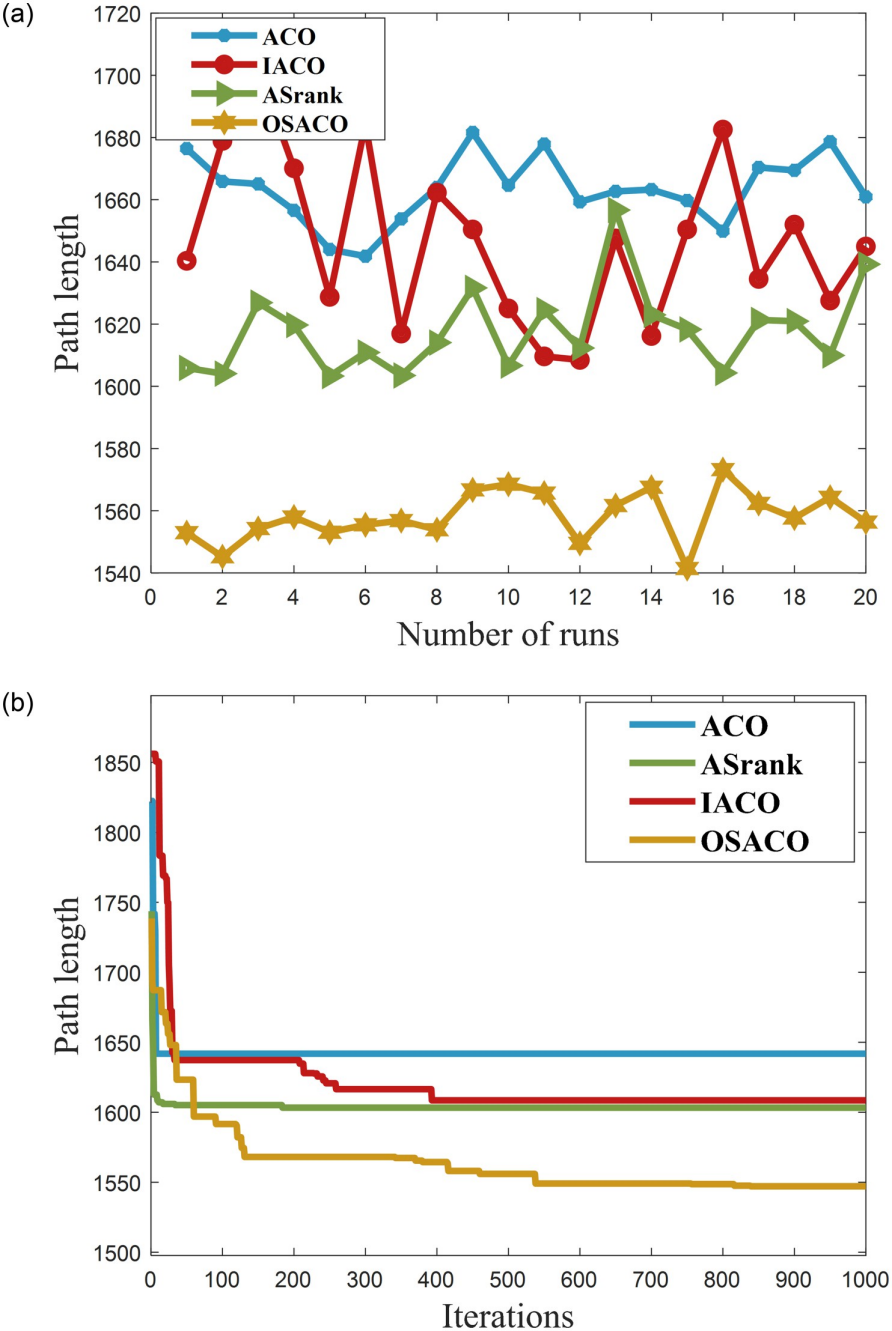
Number of independent runs and convergence curves of the four algorithms at 90 fruit numbers. (a) Four algorithms run independently for 20 times result graph. (b) Convergence curves under the optimal path conditions.

**Fig 4:**
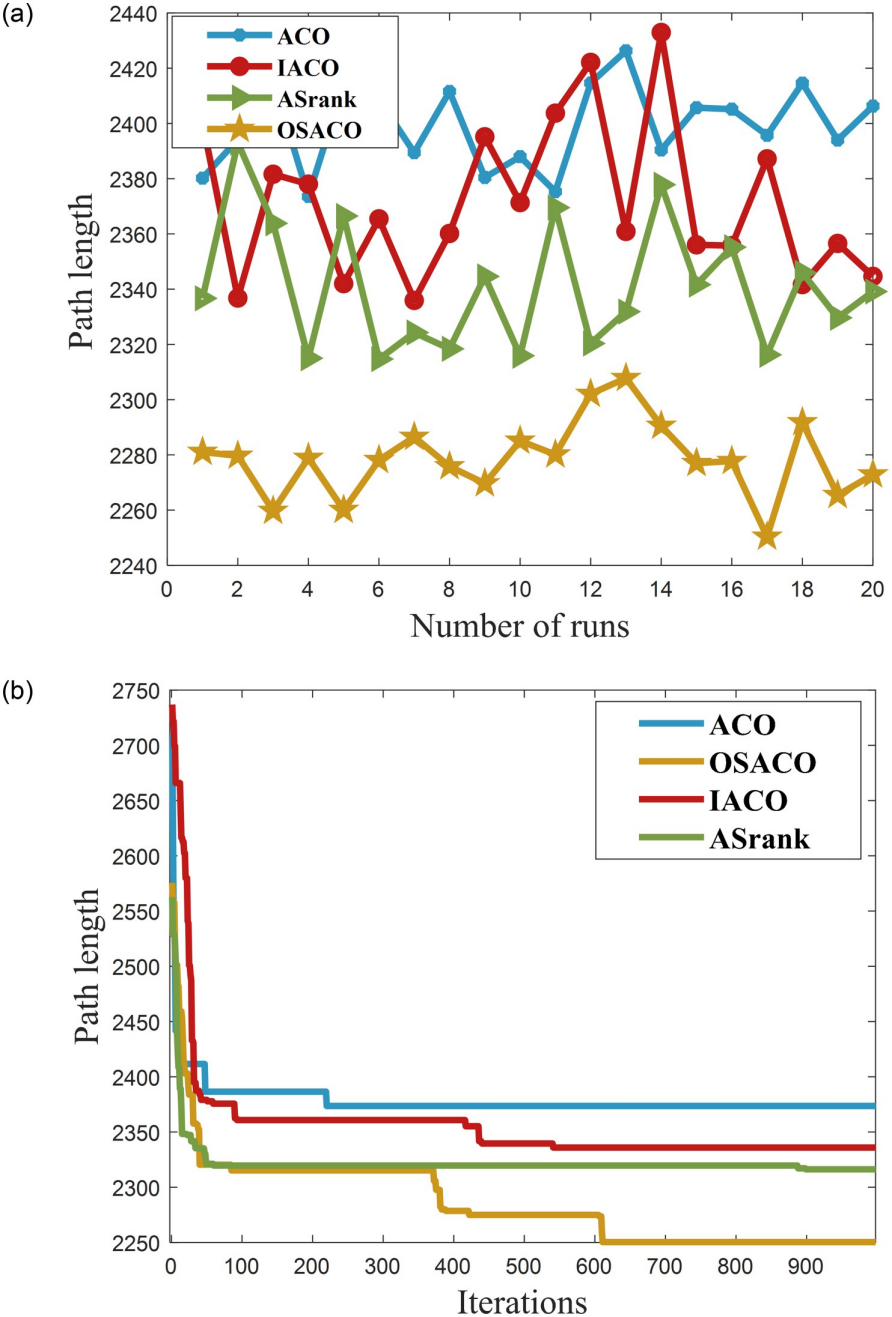
Number of independent runs and convergence curves of the four algorithms at 150 fruit numbers. (a) Four algorithms run independently for 20 times result graph. (b) Convergence curves under the optimal path conditions.

**Fig 5:**
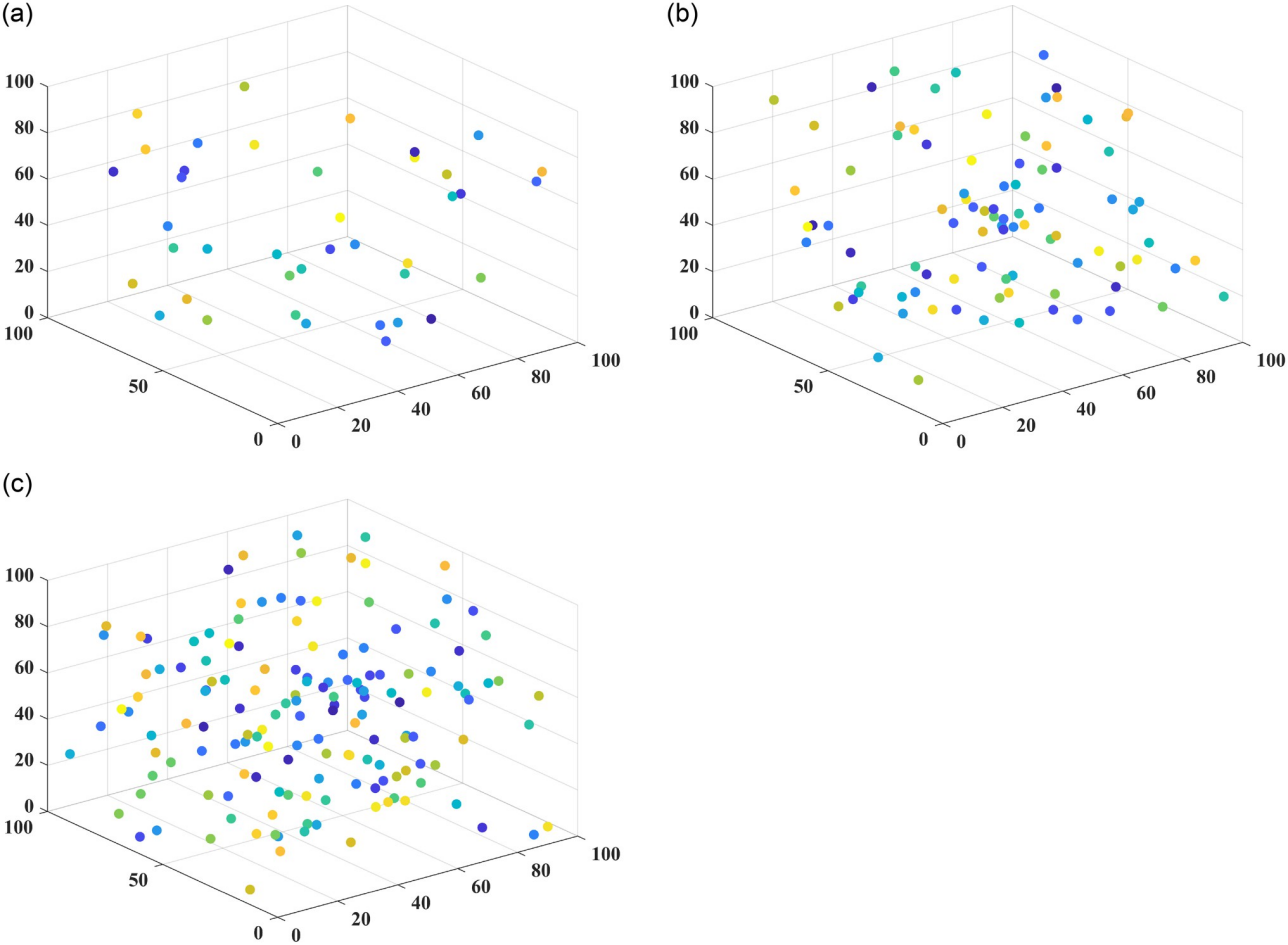
Fruit coordinate points at different scales.

**Fig 6:**
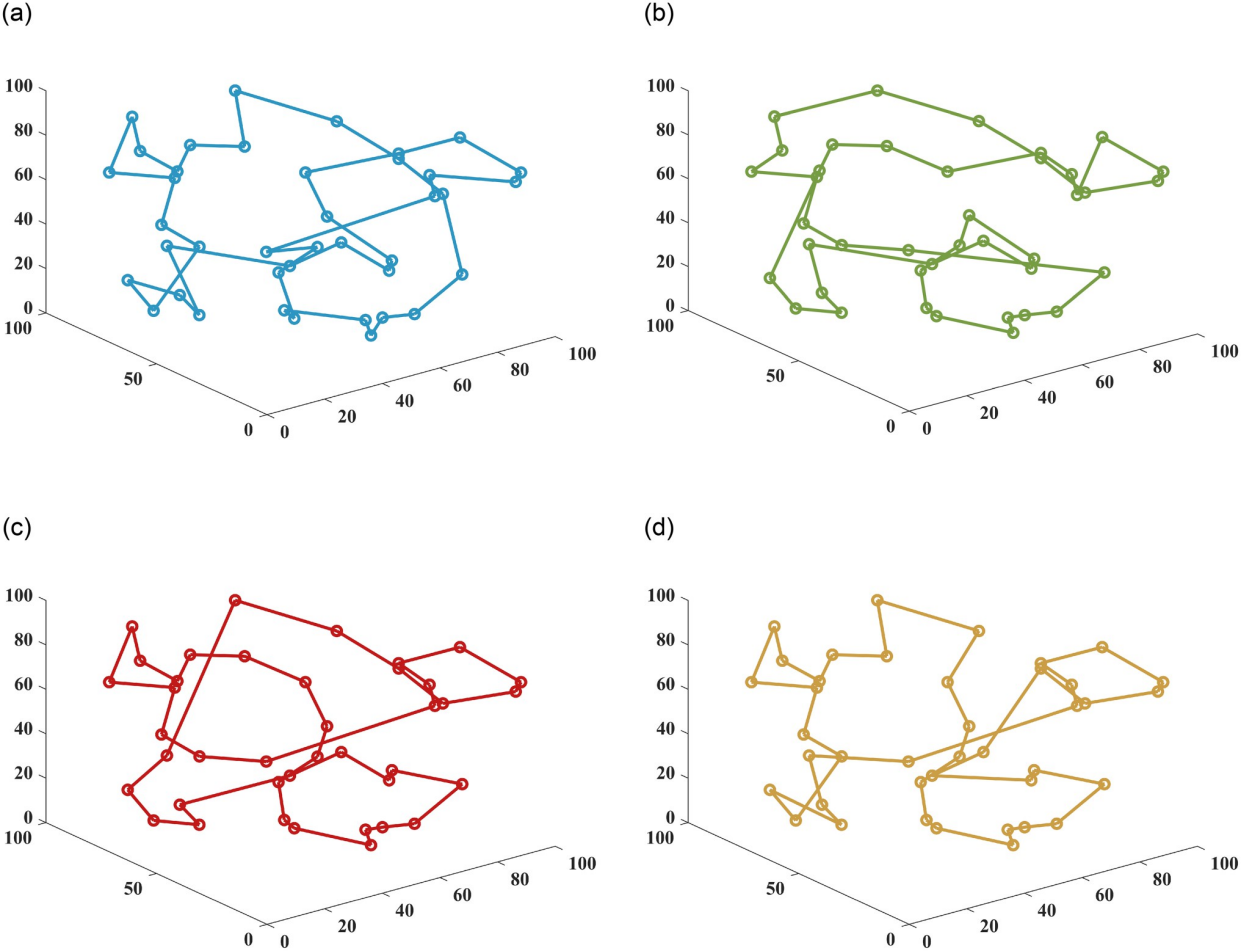
Picked roadmap obtained by four algorithms under 40 fruits. (a) ACO Picking Path. (b) ASrank Picking Path. (c) IACO Picking Path. (d) OSACO Picking Path.

**Fig 7:**
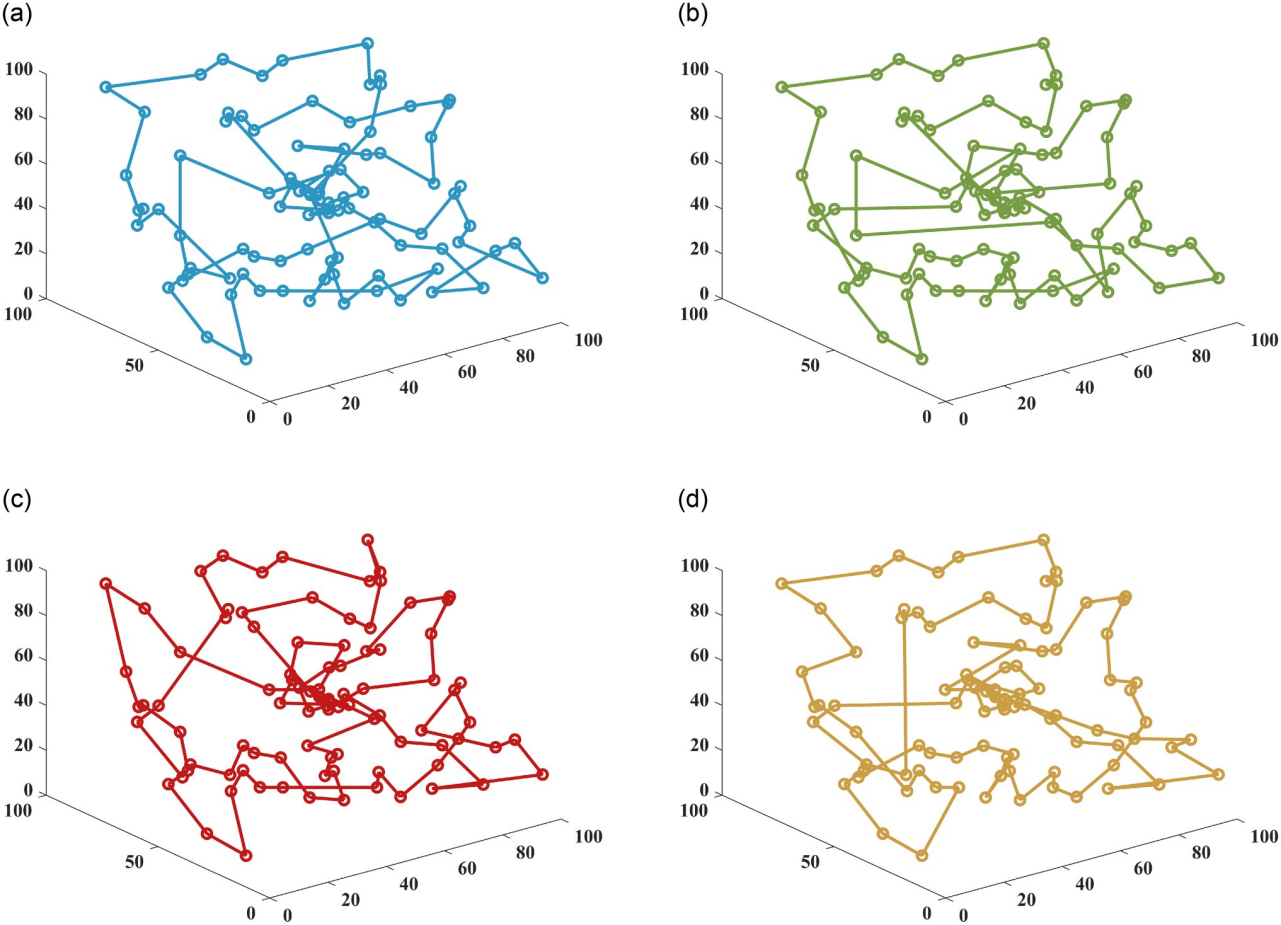
Picked roadmap obtained by four algorithms under 90 fruits. (a) ACO Picking Path. (b) ASrank Picking Path. (c) IACO Picking Path. (d) OSACO Picking Path.

**Fig 8:**
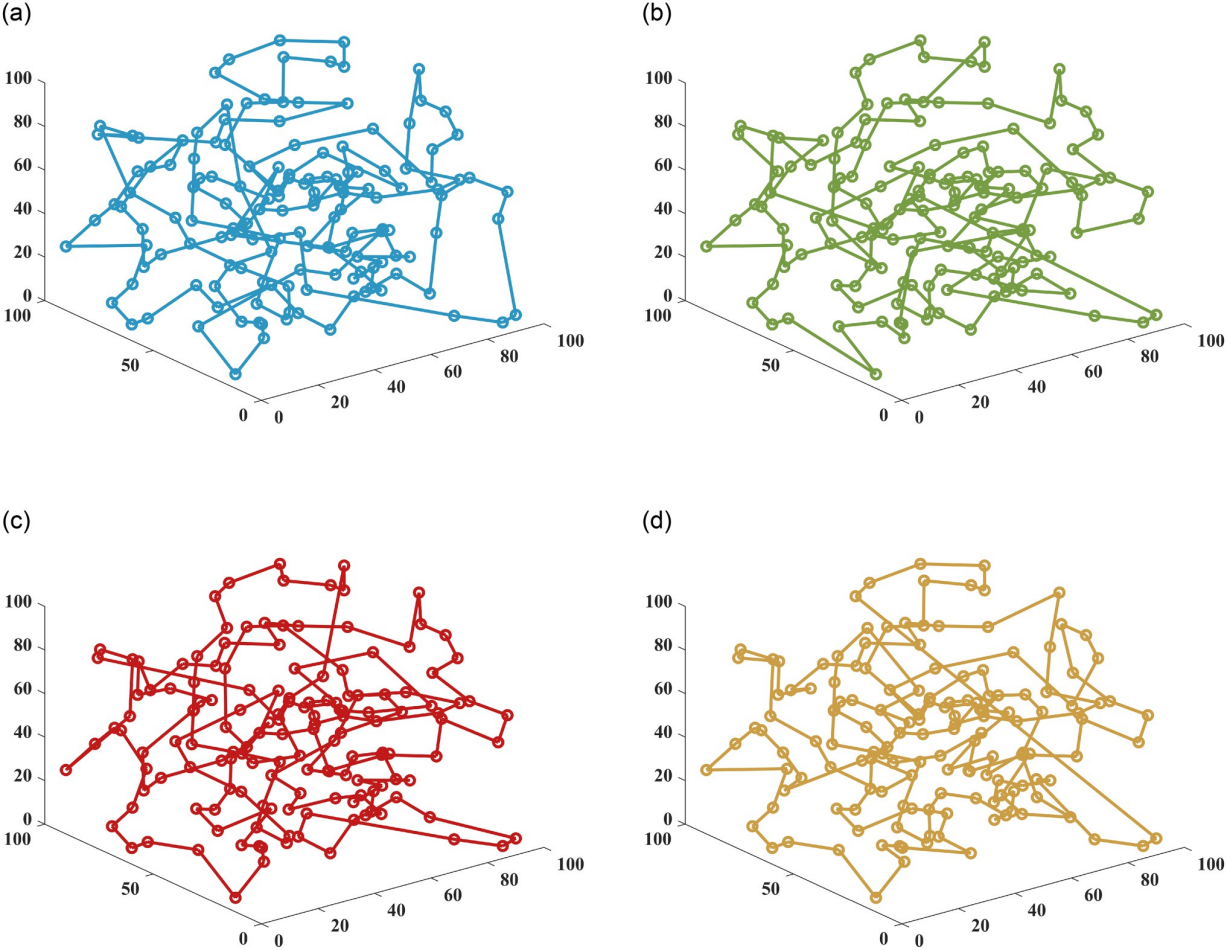
Picked roadmap obtained by four algorithms under 150 fruits. (a) ACO Picking Path. (b) ASrank Picking Path. (c) IACO Picking Path. (d) OSACO Picking Path.

In this paper, the superiority of the algorithm was verified under 40, 90 and 150 fruit conditions. The basic ant colony optimization algorithm (ACO) [4], the improved ant colony optimization algorithm (IACO) [11], and the ranking-based ant colony algorithm (ASrank) [15], three more representative ant colony algorithms, were selected for experimental comparison and analysis with the algorithm in this paper. Experimental simulation environment: 2.60GHZ main frequency Intel processor, 48G memory, simulation software is MatlabR2022a. Table 1 shows the results of the optimal parameters optimized by the multivariate adaptive genetic optimization algorithm, and Table 2 shows the results of the four different improvement algorithms at three different fruit numbers. Figs 2–4 show the results of 20 independent runs of the four algorithms under the condition of 1000 iterations at 40, 90 and 150 fruit numbers, respectively, and the convergence curves of the optimal results. Fig 5 shows the scatter plot of fruit coordinate points at three different fruit numbers. Figs 6–8 are divided into the picking roadmap of the four algorithms at three different fruit numbers.

**Table 1:** Results of each parameter obtained by optimization.

Number of fruits	Parameters			
	*α*	*β*	*Q*	*x*
40	2.4609375	8.9931641	1734.7969	25.699
90	2.302734	8.856445	387.5898	59.54102
150	1.92773	8.64453	94.7188	88.5703

**Table 2:** List of test results.

Number of fruits	Algorithm	Path Optimal Value	Path worst value	Path average	Path standard deviation
**40**	ACO	919.6231	937.0034	927.6634	5.1478
	IACO	911.5571	936.8880	916.9212	7.6899
	ASrank	863.2134	903.9489	880.0074	13.2658
	OSACO	856.4466	866.6848	860.8519	2.1997
**90**	ACO	1641.8	1681.6	1663.3	10.8547
	IACO	1608.5	1701.9	1646.7	26.7908
	ASrank	1603.3	1656.7	1617.9	13.6659
	OSACO	1541.4	1573.2	1558.2	8.0851
**150**	ACO	2373.6	2426.3	2399.2	15.1997
	IACO	2335.9	2432.9	2371.4	28.1951
	ASrank	2314.7	2393.1	2341.0	23.3450
	OSACO	2250.4	2307.8	2278.5	13.8185

As shown in Table 1, some of the initial parameters of the optimal sequential ant colony algorithm optimized by the multivariate adaptive genetic optimization algorithm for fruit numbers of 40, 90 and 150. The specific values of *α*, *β*, *Q*, *x* for different number of fruits are shown in Table 1.

As shown in Table 2, recorded are the picking path lengths for the four algorithms ACO, IACO, ASrank and OSACO at 40, 90 and 150 fruit numbers. Each algorithm was run 20 times independently for each fruit size. The best and worst paths among these 20 results are recorded, and the path means and standard deviation are calculated. Compared with the quality, optimal value, worst value, mean and standard deviation of the solutions found by the algorithms, both IACO and ASrank converge to outperform ACO at different fruit numbers. However, the standard deviation of the two is large, i.e., the algorithm is more volatile and less stable, and IACO performs more generally when the fruit numbers is small. In contrast, OSACO has a high quality of converged solutions at different scales and is highly robust.

From Figs 2(a), 3(a) and 4(a), we can see that the optimal sequential ant colony optimization algorithm is more stable and robust in solving for different fruit numbers and can find better solutions; From Figs 2(b), 3(b) and 4(b), we can see that the optimal sequence algorithm can converge to a more efficient solution regardless of the fruit number. However, the convergence speed is slightly inferior to that of the aligned ant system when the fruit number is small. From the results, the OSACO algorithm successfully overcomes the problems of premature maturity, too fast convergence, and high influence by parameters. Moreover, short continuous picking paths can be planned regardless of the number of fruits, making continuous picking much more efficient. Compared with the ant colony algorithm and its variants, OSACO maintains a better convergence rate of the algorithm when the number of fruits is larger, and the quality of the solutions converged to is higher.

The 3D coordinate points of the fruit were visualized for a more intuitive understanding of the picking path map, and the results of the fruit coordinate visualization are shown in Fig 5.

Figs 6–8 show the results of the optimal picking path visualization obtained by the four algorithms for the number of 40, 90 and 150 fruits. According to Table 2, the OSACO algorithm obtains the shortest picking path regardless of the number of fruits. If we look closely at the case, we will find that OSACO algorithm also obtains fewer path transitions. Such visualization diagrams also allow us to better understand how the three-dimensional continuous picking process works.

## Conclusion

The purpose of this study is to obtain a new algorithm to plan the successive picking paths of a picking robot, thus improving the efficiency of fruit picking. In this paper, the path planning problem of continuous fruit picking is transformed into a three-dimensional TSP problem without considering obstacle avoidance, and an OSACO algorithm is proposed. Taking the coordinate nodes of each three-dimensional fruit as the coordinates of the three-dimensional city in the TSP problem, the optimal path for continuous fruit picking is obtained under the condition that all the fruit coordinate nodes are traversed and one node can only be reached once. The OSACO algorithm is compared with the ACO algorithm and its improved algorithm. The experimental results showed that the average path length of OSACO at 40 fruit number scale was 92.79% of ACO, 93.89% of IACO, and 97.82% of ASrank; the average path length of OSACO at 90 fruit number scale was 93.68% of ACO, 94.63% of IACO, and 96.31% of ASrank; the average path length of OSACO at 150 fruit number scale was 94.97% of ACO, 96.08% of IACO, and 97.33% of ASrank. OSACO reduces the average path length by 6.18% compared to the ACO algorithm, 2.9% to ASrank, and 5.2% to IACO. However, the standard deviation of these three algorithms is high, the algorithm fluctuates, the robustness is not high, and the convergence effect is unstable. From the simulation experimental results, it can be seen that OSACO algorithm is much better than ACO and its improved algorithm in terms of solution accuracy, convergence stability and algorithm robustness, which will enable the picking robot to complete the picking work with a shorter continuous picking path, which will also lead to a great improvement in its working efficiency. The significance of this paper is that the OSACO algorithm is proposed and applied to 3D continuous picking path planning, which allows us to obtain shorter continuous picking paths more consistently, thus significantly improving the picking efficiency of the picking robot.

## Limitations and future works

In this paper, the research on fruit picking robot picking path can be applied to many occasions, like apple, citrus, lychee, and even tomato picking, which has substantial application value. While our work is a meaningful step towards continuous fruit-picking path planning, it also has several limitations. Firstly, it is assumed that all fruit-picking points are known; secondly, obstructions such as tree branches are not considered. This paper only provides a novel approach to the path planning aspect of continuous picking for fruit-picking robots, which still needs to be optimized and improved in practical applications.

Deep learning is a hot research topic nowadays, so our future work will combine with deep learning to make the picking robot with visual recognition function and high picking efficiency at the same time. The fruit-picking robot can obtain the three-dimensional coordinates of ripe fruits and plan an optimal continuous picking path considering obstacles such as tree branches. In the future, we will continue to improve and optimize its performance in practical applications to make the picking robots more intelligent and functionally integrated, thus promoting agricultural modernization and improving agricultural efficiency.

## Supporting information

S1 Data I have uploaded a supporting information file. The file name is “Data for Three-dimensional continuous picking path planning based on ant colony optimization algorithm”.
(ZIP)
